# Postpartum care for Aboriginal and non-Aboriginal women with Gestational Diabetes Mellitus across urban, rural and remote locations: a protocol for a cohort linkage study

**DOI:** 10.1186/2193-1801-2-576

**Published:** 2013-10-30

**Authors:** Catherine Chamberlain, Bronwyn Fredericks, Bronwyn Davis, Jacqueline Mein, Catherine Smith, Sandra Eades, Brian Oldenburg

**Affiliations:** Global Health and Society Unit, Department of Epidemiology and Preventive Medicine, Faculty of Medicine, Nursing and Health Sciences, Monash University, L3/89 Commercial Rd, Prahan, Victoria 3181 Australia; Office of Indigenous Engagement, Central Queensland University, Bruce Highway, North Rockhampton, Queensland 4701 Australia; Cairns Diabetes Centre, PO Box 902, Cairns, Queensland 4870 Australia; Apunipima Cape York Health Council, 186 McCoombe Street, Cairns, Queensland 4870 Australia; Department of Epidemiology and Preventive Medicine, Faculty of Medicine, Nursing and Health Sciences, Monash University, L6/96 Commercial Rd, Prahan, Victoria 3181 Australia; Sydney School of Public Health, Sydney Medical School, The University of Sydney, Sydney, New South Wales 2006 Australia

**Keywords:** Gestational diabetes mellitus, Type 2 diabetes mellitus, Diabetes, Pregnancy, Aboriginal, Indigenous

## Abstract

**Background:**

Gestational diabetes mellitus (GDM) is increasing, along with obesity and type 2 diabetes (T2DM), with Aboriginal and Torres Strait Islander (Aboriginal^a^) women in Australia particularly affected. GDM causes serious complications in pregnancy, birth, and the longer term, for women and their infants. Women with GDM have an eightfold risk of developing T2DM after pregnancy, compared to women without GDM. Indigenous women have an even higher risk, at a younger age, and progress more quickly from GDM to T2DM, compared to non-Indigenous women. If left undetected and untreated, T2DM increases risks in subsequent pregnancies, and can lead to heart disease, stroke, kidney failure, limb amputations and blindness for the woman in the longer term. A GDM diagnosis offers a ‘window of opportunity’ to provide acceptable and effective prevention, treatment, and postpartum care. Low rates of postpartum T2DM screening are reported among non-Aboriginal women in Australia and Indigenous women in other countries, however, data for Aboriginal women in Australia are scarce. A healthy diet, exercise and breastfeeding can delay the onset of T2DM, and together with T2DM screening are recommended elements of postpartum care for women with GDM. This paper describes methods for a study evaluating postpartum care among Aboriginal and non-Aboriginal women with GDM.

**Methods/Design:**

This retrospective cohort includes all women who gave birth at Cairns Hospital in far north Queensland, Australia, from 2004 to 2010, coded as having GDM in the Cairns Hospital Clinical Coding system. Data is being linked with the Midwives Perinatal Data Collection, and the three local laboratories. Hospital medical records are being reviewed to validate accuracy of GDM case ascertainment, and gather information on breastfeeding and provision of dietary advice. Survival analysis is being used to estimate time to screening, and rates of progression from GDM to T2DM. Logistic regression is being used to compare postpartum care between Aboriginal and non-Aboriginal women, and assess factors that may be associated with provision of postpartum care.

**Discussion:**

There are challenges to collecting postpartum data for women with GDM, however, this research is urgently needed to ensure adequate postpartum care is provided for women with GDM.

**Electronic supplementary material:**

The online version of this article (doi:10.1186/2193-1801-2-576) contains supplementary material, which is available to authorized users.

## Background

Gestational diabetes mellitus (GDM) is increasing along with obesity (Hunt and Schuller 2007) and type 2 diabetes mellitus (T2DM) (Sicree et al. 2009; Australian Institute of Health and Welfare 2010b), with Indigenous^a^ populations particularly affected (Naqshbandi et al. 2008). GDM causes serious complications in pregnancy, birth (HAPO Study Cooperative Research Group 2008; Coustan et al. 2010) and the longer term (Dyck et al. 2010), for both women and their infants. Compared to non-Indigenous women, Indigenous women have a higher risk of GDM (Steinhart et al. 1997; Dyck 2005; Young et al. 2002), at a younger age (Yue et al. 1996; Benjamin et al. 1993), and there is a much higher rate of both diagnosed and undiagnosed T2DM in pregnancy (Australian Institute of Health and Welfare 2010b). Women diagnosed with GDM have a very high risk of developing T2DM postpartum, compared to women who do not have GDM (Kim 2002; Bentley-Lewis et al. 2008; Chodick et al. 2010; Bellamy et al. 2009; Heikes et al. 2008), and Indigenous women experience the highest risk (Yue et al. 1996; Kim 2002). The emergence of diabetic disorders among young child-bearing women represents an ominous ‘tipping point’ (Canadian Diabetes Association 2011) in the diabetes epidemic (Yue et al. 1996), as exposure to diabetes in-utero also significantly compounds the health risks for the next generation (Dyck et al. 2010; Osgood et al. 2011), and GDM becomes an additional driver for T2DM (Bhattarai 2009; Osgood et al. 2011). T2DM is a serious metabolic disorder, characterised by hyperglycaemia and, if left undetected and untreated, increases the risk of serious complications in subsequent pregnancies, including congenital abnormalities (Bower et al. 1992; Farrell et al. 2002), and can lead to heart disease, stroke, kidney failure, limb amputations and blindness (Australian Institute of Health and Welfare 2010a). T2DM is a major cause of death and disability among Aboriginal people (Australian Bureau of Statistics 2008) and directly contributes to health disparities in Australia (Australian Bureau of Statistics 2010).

GDM includes pre-existing diabetes that has not been diagnosed before pregnancy, or temporarily glucose intolerance expedited by growth hormones in pregnancy (American Diabetes Association 2009). The increased insulin demands of pregnancy can ‘unmask’ (Lee et al. 2007) abnormalities in beta-cell function (Brown and Trost 2003; Moran et al. 2010), forewarning of the risk of progression to T2DM (Bilhartz et al. 2011). Most importantly, it offers a unique ‘window of opportunity’ for public health strategies because young women with no other identified conditions usually have frequent scheduled contacts with health-care providers for pregnancy care, often for the first time since early childhood. Pregnant women are also often highly motivated to adapt their lifestyles to improve the health of their infant (Kalra et al. 2011), with any effective support potentially benefitting the whole family (McBride et al. 2003; Orleans et al. 2000). The postpartum period also offers unique opportunities for women to reduce their risk (Schwarz et al. 2010; Liu et al. 2010) and the long-term risk for their infant (Owen et al. 2006; Pettitt et al. 1997), through breastfeeding.

Evidence about the risks of GDM (Coustan et al. 2010) has led to changes to international (International Association of Diabetes and Pregnancy Study Groups 2010) and national screening guidelines (Teh et al. 2011; Nankervis et al. 2013). The major changes include: offering screening in early pregnancy for women at high risk of T2DM, in addition to 24–28 weeks’ as is currently recommended; separating ‘probable’ undiagnosed T2DM from GDM; and changing the diagnostic thresholds for GDM. These changes are likely to significantly increase the prevalence of GDM in Australia (Round et al. 2010; Moses et al. 2011; Lindsay 2011; Morikawa et al. 2010; Leiberman et al. 2011; Flack et al. 2010; O’Sullivan et al. 2011), and have particular implications for Aboriginal^a^ women, who are categorised as having a high risk of T2DM (Chamberlain et al. 2011). While there are potential benefits, there are key criteria for introducing population-based screening, which specify that the benefits must outweigh the risks and inconvenience (Wilson and Jungner 1968), and that effective prevention, treatment and follow-up (postpartum) are provided (Australian Health Ministers’ Advisory Council 2008).

Despite the clear evidence of an increased risk of developing T2DM (Steinhart et al. 1997; Dyck et al. 2010; Young et al. 2002; McGrath et al. 2007), there are few studies investigating rates of postpartum T2DM screening for Aboriginal women with GDM (Chamberlain et al. 2013). Low rates of postpartum screening for T2DM have been reported for non-Indigenous women in Australia (Russell 2006; Morrison et al. 2009; Kim 2007; Sterne et al. 2011) and internationally (Pierce et al. 2011; Tovar et al. 2011; Keely et al. 2010), as well as Indigenous women in Canada (Shah et al. 2011; Mohamed and Dooley 1998), New Zealand (McGrath et al. 2007) and the United States (Steinhart et al. 1997). A review of postpartum diabetes screening reported rates ranging from 34% to 73%, with marked variations by race/ethnicity (Tovar et al. 2011b). One study reported low rates of postpartum screening for Aboriginal women in far north Queensland, however the region is confined to remote areas only and numbers were too small to assess trends (Davis et al. 2013). These low rates of postpartum T2DM screening are in stark contrast to high rates of postpartum screening for cervical cancer (Sterne et al. 2011), with one study reporting only 37% of eligible women underwent a postpartum Oral Glucose Tolerance Test (OGTT), while 94% underwent a postpartum papanicolaou test (Smirnakis 2005), which is also perceived as an unpleasant test for many women.

Some of the factors reported as barriers to postpartum screening include; lack of awareness of the need to attend screening, the inconvenience of the OGTT (which requires fasting, consuming a glucose drink, and a number of blood tests over several hours), and the need to attend with small children (Sterne et al. 2011; Bell et al. 2011; Clark and Keely 2012). However, there are likely to be additional barriers for women living in rural and remote areas (Eades et al. 2010). Rural and remote communities face challenges accessing health services due to the rugged and sometimes inaccessible terrain, and they may be required to travel long distances to access specialist services, including an OGTT. However, local services are not likely to incur fees, and individuals are more likely to be personally known to service providers in small communities. Most Aboriginal people now reside in urban areas, where there is comparatively limited research, particularly ‘intervention research’ (Eades et al. 2010), and different barriers which are not well understood (Eades et al. 2010). There may be limited publicly funded health services in regional urban areas, and private services may incur substantial fees. While there may be administrative arrangements to cover costs for health care card holders and/or Aboriginal women, these arrangements may not be well understood by women or healthcare providers in urban areas. While research suggests relatively simple strategies can increase postpartum diabetes screening (Carson et al. 2013), such as: structured systems (Mohamed and Dooley 1998), proactive postpartum care plans (Gabbe et al. 2011), antenatal education (Stasenko et al. 2011), physician reminders (Lega et al. 2011), patient reminders (Korpi-Hyovalti et al. 2012) and registers (Dannenbaum et al. 1999), local circumstances will need to be considered.

Effective lifestyle and breastfeeding support has been shown to reduce the risk of T2DM for non-Indigenous women and their children during (Landon et al. 2009; Crowther et al. 2005) and after pregnancy (Knowler 2002; Tuomilehto 2001; Pan 1997; O’Reilly et al. 2011; van der Pligt et al. 2013). There are no studies reporting effective diet and exercise support for Aboriginal women with GDM (Dyck et al. 1998; Gray-Donald et al. 2000; Klomp et al. 2003; Chamberlain et al. 2013), and studies report a lower sense of self-efficacy about postpartum weight loss among women categorised as ‘low socioeconomic status’ (SES). Breastfeeding support has also been shown to be effective (Karanja et al. 2010) and feasible (Murphy and Wilson 2008) among Indigenous women in the United States (US) and Canada, however no studies have been reported in Australia, despite one study suggesting breastfeeding rates may be lower among Aboriginal women with GDM than those without (Simmons et al. 2005). This is not surprising given women with GDM and their infants are more likely to experience complications which may inhibit breastfeeding (e.g. caesarean section, neonatal hypoglycaemia), and evidence these complications are more likely to have a differential impact on women categorised as ‘low SES’ , as they are more likely to have lower self-confidence and sense of self-efficacy about their ability to breastfeed (Demirtas 2012). While broader strategies addressing environmental determinants have been suggested (O’Dea et al. 2007; Stephenson 1993; Young et al. 2002), no such strategies have yet been reported (Chamberlain et al. 2013). The paucity of good quality ‘intervention research’ to prevent T2DM among Indigenous peoples has similarly been reported (McNamara et al. 2011), highlighting a gap in diabetes research more generally.

Adequate support for women diagnosed with GDM is also important for psychological wellbeing. The diagnosis of any medical condition can be associated with increased psychological stress (Sable and Wilkinson 2000), particularly during pregnancy as women are concerned about the health of their infant (Daniells et al. 2003; Rumbold and Crowther 2002; Langer and Langer 1994; Cosson 2010). While recent studies suggest that a GDM diagnosis may not increase stress among non-Aboriginal women in Australia (Coustan 2010; Rumbold and Crowther 2002), it has been reported among ethnic minority groups (Razee et al. 2010). Furthermore, studies among Indigenous women in the US and Canada, report increased stress (Neufeld 2011) and ‘risk perception’, coupled with low perceptions of ‘self-efficacy’ associated with a GDM diagnosis, despite high levels of knowledge (Jones et al. 2012). Aboriginal women may be more likely to experience additional stressors. For example, they are more likely to live in geographically remote areas and may be required to travel to a major urban city for specialist care and be thousands of miles from family, and they are more likely to know people experiencing serious consequences of T2DM. Effective interventions and postpartum care are critical to improving confidence and self-efficacy with regards to lifestyle and breastfeeding, reducing stress associated with a diagnosis of GDM, and providing appropriate treatment when needed to mitigate the risks to subsequent pregnancies and the long term risks for women.

### Research aims and objectives

This paper describes the methods for a retrospective cohort study which aims to evaluate postpartum care for Aboriginal and non-Aboriginal women with GDM in urban, rural and remote regions in far north Queensland from 2004 to 2010. The purpose is to identify barriers and enablers to improve postpartum care for women with GDM, in all geographic regions. Trend analysis over time is also essential for evaluating whether improvements are occurring as changes have been introduced.

More specifically, the objectives are to:

1. report the proportion of women diagnosed with GDM who receive postpartum T2DM screening as per guidelines (OGTT at 6 weeks, annually and biannually thereafter) (Queensland Health and Royal Flying Doctors Service (Queensland Section) 2009);
2. report the rate of progression from GDM to T2DM;
3. investigate the recorded rates of other preventive activities, such as breastfeeding and visits to a dietician or diabetes educator during pregnancy; and
4. assess the degree to which confounders impact on rates of postpartum T2DM screening, progression to T2DM, breastfeeding, and dietician or diabetes educator consultations.

## Methods

### Study design and setting

**Figure 1:**
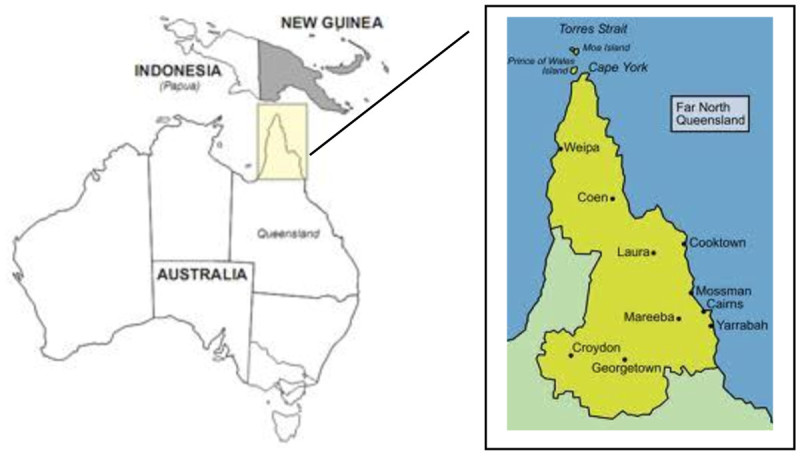
**Far North Queensland.** Source: (Wikipedia 2013).

The study is being conducted in far north Queensland, a vast region covering almost 300,000 square kilometres on the north east tip of Australia (See Figure 1), excluding the Torres Strait Islands. The region has a population of over 230, 000 people, and approximately 40,000 (17%) Aboriginal and Torres Strait Islander people (Queensland Health 2008). About half of the population live in the main regional (urban) centre of Cairns, which is approximately 1750 kilometres north of the main metropolitan centre (Brisbane) in Queensland. About 50% of people in this region live in areas classified as rural and remote, in a sparsely populated tropical region which has limited sealed road access and is subject to extreme weather events, which may make areas even less accessible during some seasons.

In far north Queensland, there have been a number of initiatives introduced over the past decade to improve care for women with GDM. An audit of screening practice and outcomes *during* pregnancy (Davis et al. 2009) has led to introduction of a protocol (Queensland Health and Royal Flying Doctors Service (Queensland Section) 2009; 2005; 2007) which includes recommendations for postpartum T2DM screening, promoting breastfeeding, and providing lifestyle advice. This protocol was introduced in the Torres Strait Islands in 2000 and in the Cape, an area to the far north of the Australian mainland, since 2006.

The study cohort is being recruited from Cairns Hospital (CH), which is the public hospital referral centre in far north Queensland. The CH catchment area includes Cairns and hinterland, Cape York and the Torres Strait Islands. CH provides maternity services for over 80% of women in far north Queensland, with the remaining women attending private or other small local public hospitals. It is recommended that all women with diabetes requiring insulin therapy in pregnancy are referred to CH. During the study period (1/1/2004–31/12/2010), the diagnostic criteria for GDM were consistent with the Australian Diabetes in Pregnancy Society guidelines (Hoffman et al. 1998), which required fasting plasma glucose (FPG) > =5.5 mmol/L or two-hour glucose > =8 mmol/L following a 75 g oral glucose tolerance test (OGTT). However, the standard procedure for GDM screening varied markedly across Australia during this period (Depczynski et al. 2011), including in far north Queensland (Davis et al. 2013; 2009). In 2005, primary care guidelines recommended a random blood glucose level (RBG) at each pregnancy visit, and if the level was > =5.0 mmol/L, a fasting blood glucose level (FBG) was offered (Queensland Health and Royal Flying Doctors Service (Queensland section) 2005). At 24 weeks, a RBG was offered again and if > =5.0 mmol/L or the woman was classified as ‘at risk’ of GDM (Aboriginality was not a risk factor), a 75 g OGTT was offered. If normal, the 75 g OGTT was to be offered again at 32 weeks. Glycosylated Haemoglobin (HbA1c) was supposed to be routinely offered around 28 weeks or at first presentation. In 2007, all Aboriginal^a^ women were classified as ‘high risk’ and advised to have a 75 g OGTT at 26–30 weeks gestation (Queensland Health and Royal Flying Doctors Service (Queensland section) 2007). In 2009 the guidelines were revised to recommend a 75 g OGTT at 24 to 28 weeks gestation (Queensland Health and Royal Flying Doctors Service (Queensland Section) 2009), as per the Australian Diabetes in Pregnancy Society guidelines (Hoffman et al. 1998). GDM screening rates were estimated to be 99.5% on the Torres Strait Islands in 2006 (Falhammar et al. 2010). Among Aboriginal women in Cape York, screening rates ranged from 31.4% in 2006 to 65.6% 2008, with a subsequent increase in diagnosis of GDM from 4.7% in 2006 to 14.7% in 2008 (Rumbold et al. 2011; Davis et al. 2013).

### Data sources

The study is using linked electronic data, with key variables validated by a sample of medical record reviews. Data is being collected from four main sources: (1) the CH Clinical Coding (CHCC) system; (2) Midwives Perinatal Data Collection (MPDC); (3) the three laboratories in far north Queensland, and (4) CH medical records.

1. *Cairns Hospital Clinical Coding (CHCC) system*
  The study population has been identified from the CHCC system. This coding is done by administrative staff, based on what is documented in the medical records for each inpatient episode. This data is primarily collected for funding purposes, though may be used in quality improvement and other research initiatives. All episodes of care are recorded and the reason for care classified under the International Classification of Diseases (ICD) codes.

(2) *Midwives Perinatal Data Collection (MPDC)*
  The Queensland Health Statistics Unit collates the MPDC data collected by clinicians at birth. This data is collected separately from any administrative data collection processes and is a legal requirement for all ‘birth attendants’ in Australia. All data collected are items which are considered important for monitoring perinatal health status and healthcare at state and national levels. Data received from midwives is entered into the MPDC with a range of mechanisms to ensure data quality, including blinded double data entry, to reduce data entry error. The MPDC form is based on the National Health Data Dictionary (Australian Institute of Health and Welfare 2012), and although there have been variations between Australian states and territories over time (Laws and Sullivan 2004), validation studies in other states show high sensitivity (>95%) for collecting GDM data (Taylor et al. 2005; Metcalfe 2012).

(3) *Laboratory data*
  Postpartum OGTT screening and diagnosis is exclusively undertaken in the three local laboratories (one public and two private) in far north Queensland. Laboratories also conduct other glucose screening tests (HbA1C, FPG, RPG), which can be provided by many healthcare providers in the region. Private laboratories are providing data on the type of T2DM screening test, date of test, whether during pregnancy or not (to exclude pregnancy screening), and test results. The public laboratory data is being extracted onto a database by clinical members of the research team who are authorised to access patient information.

(4) *CH Medical Record Review*
  Data from CHCC and MPDC is being linked on a spread-sheet and downloaded into a Microsoft Access database. A random sample of medical records of women with GDM are being reviewed to ensure accuracy of case identification and confinement date, complete missing electronic data fields, and collect some additional data not available elsewhere (for example, antenatal and postnatal care provider type, number of dietician and diabetic educator consultations, indication for induction or caesarean section, provision of artificial infant formula). The medical record review is being conducted by a registered midwife. The first 10 medical records are being reviewed by both staff together to ensure consistency of collection and 100 records co-reviewed to assess inter-rater reliability.

Data on postpartum screening from the Primary Health Care Information System (PHCIS), used by many public primary health care providers in far north Queensland, has been examined but is not able to be extracted in a usable format for this study. Primary health care services would not be able to provide OGTTs, which are the recommended screening and diagnostic test for T2DM screening and the subject of this study. However there may be some ‘point of care’ tests that will not be included in this study, such as HbA1C, FPG, and RPG, which were provided by primary health care providers. Postpartum screening rates will be reported as proportions who received the recommended screening tests (OGTT), or ‘Any postpartum glucose screen’. The limitations about the rates of ‘any’ postpartum glucose screens in the study will be reported, with recommendations for future prospective studies to collect these data.

### Sample size calculation

A sample size of 325 Aboriginal and 325 non-Aboriginal women has been calculated to be 80% powered to detect a 10% difference in women receiving postpartum screening for T2DM, with a 95% confidence interval. These figures were based on consultation with experienced clinical staff, who estimated that approximately 20% of Aboriginal women and 30% of non-Aboriginal women currently receive postpartum T2DM screening. Whether there is a significant difference will help to ascertain whether some of the barriers or enablers to postpartum care may be culturally-specific, or generic for all women with GDM.

### Study sample and case ascertainment

The study includes all women who gave birth at CH between 1/1/2004 to 31/12/2010 and have an appropriate GDM ICD-10-AM code assigned (024.41, 024.42, 0.24.43, 0.24.44) in the CHCC and MPDC (Australian Institute of Health and Welfare 2010b). Women with an ICD code indicating pre-existing diabetes (024.0, 024.11, 024.12, 024.13, 024.14, 024.21, 024.22, 024.31, 024.32, 024.9) are being excluded (see Table 1). The sample will include electronic records for 353 Aboriginal women and 659 non-Aboriginal women, therefore the study will be adequately powered to test the estimated 10% difference in postpartum screening.

**Table 1:** **Diabetes in pregnancy International Classification of Disease (ICD) codes**

ICD code	Description	Included in this study
**O24.0**	Pre-existing diabetes mellitus, Type 1, in pregnancy	No
**024.11**	Pre-existing diabetes mellitus, Type 2, in pregnancy, non-insulin treated	No
**024.12**	Pre-existing diabetes mellitus, Type 2, in pregnancy, insulin treated	No
**O24.13**	Pre-existing diabetes mellitus, Type 2, in pregnancy, oral hypoglycaemic therapy	No
**O24.14**	Pre-existing diabetes mellitus, Type 2, in pregnancy, other	No
**024.21**	Pre-existing diabetes mellitus, other specified type, in pregnancy, non-insulin treated	No
**024.22**	Pre-existing diabetes mellitus, other specified type, in pregnancy, insulin treated	No
**024.31**	Pre-existing diabetes mellitus, unspecified, in pregnancy, non-insulin treated	No
**024.32**	Pre-existing diabetes mellitus, unspecified, in pregnancy, insulin treated	No
**O24.41**	Diabetes mellitus arising at or after 24 weeks gestation, non-insulin OR Diabetes mellitus arising in pregnancy, non-insulin-requiring	Yes
**O24.42**	Diabetes mellitus arising at or after 24 weeks gestation, insulin treated OR Diabetes mellitus arising during pregnancy, insulin treated OR Diabetes mellitus arising in pregnancy, insulin-requiring	Yes
**O24.43**	Diabetes mellitus arising during pregnancy, oral hypoglycaemic therapy	Yes
**O24.44**	Diabetes mellitus arising during pregnancy, other	Yes
**024.9**	Diabetes mellitus in pregnancy, unspecified onset	No

Source: Diabetes in pregnancy: its impact on Australian women and their babies (Australian Institute of Health and Welfare 2010b).

The quality of retrospective data in this study is being improved by use of data linkage, and a review of a sample medical records to ensure key variables are correct. The sample selected for review includes medical records of all Aboriginal (n = 353), all non-Aboriginal women living in remote areas (n = 13), and a random sample of 236/659 (36%) non-Aboriginal women. The random sample has been selected using a computer-generated random number sequence.

### Outcome assessment

Primary outcomes for this study include the time to postpartum OGTT or any laboratory-based glucose test, and the proportions screened (OGTT or any) according to recommended protocols:

- the proportion of women with GDM who received; *all* postpartum OGTTs as per protocol
- times to screening test (OGTT or any)
- cumulative rates of OGTT or ‘any’ postpartum glucose test at 6 weeks (0–6 months), 12 months (6–24 months) and 3 years (24–48 months)
- the proportion of GDM pregnancies who received an OGTT or any postpartum glucose test at 6 weeks (0–6 months), 12 months (6–24 months) and 3 years (24–48 months), excluding tests providing in the previous period.

Women will be censored from ‘screening eligibility’ from:

- time of onset of subsequent pregnancies, calculated as 273 days prior to subsequent pregnancy, or 20 weeks prior to date of test if test after 1/3/2010, and coded as ‘during pregnancy’ (to capture possible confinements after 31/12/2010).
- date of type 2 diabetes diagnosis, calculated if the records from any datasource (e.g. medical records, MPDC data, laboratory tests) indicated the women developed type 2 diabetes. If a type 2 diabetes diagnostic date is not available, the date of the glucose screening test will be used as the censorship date.

All screening test results for each woman with GDM are being assessed by a registered midwife on the research team. The assessment includes (1) whether some or all of the criteria for follow-up screening were met (see Table 2), (2) whether further follow-up screening is recommended (3) date of last screening test (4) date of diagnosis of T2DM and test used for diagnosis, and (5) months from date of confinement to screening. The records will be reviewed by an expert diabetes clinician where it is unclear whether further screening is recommended.

**Table 2:** **Criteria used to assess adequacy of postpartum T2DM screening for women with GDM**

1.	Diagnosed with GDM in last pregnancy only (not any previous pregnancy) and:	
	● Is more than 6 weeks but less than 6 months postpartum and has not had a ‘6 week’ OGTT;	
	● Is more than 6 months postpartum but less than 2 years and has not had a ‘1 year’ OGTT;	
	● Is more than 2 years postpartum and previously identified ‘impaired glucose tolerance’ on a*ny* test (OGTT, FPG, HBA1C or RPG), and has not had an OGTT in the past year.	
	● Is more than 3 years postpartum and has not had an OGTT in the last 2 years	
	● Had a FPG or RPG on any of the above occasions which was within the ‘impaired’ or ‘abnormal’ glucose tolerance range (as outlined in Table 3) and did not have an OGTT.	
2.	Diagnosed with pre-existing T2DM in pregnancy and no record of any postpartum tests	

Secondary outcomes include:

1. proportion of women with ‘probable’ T2DM (diagnosis prior to 16 weeks gestation) who received a postpartum OGTT;
2. rates of follow-up screening for women identified as having impaired glucose tolerance (OGTT annually);
3. progression time from GDM to T2DM diagnosis;
4. proportion of women with GDM for whom an appointment with a dietician or diabetes educator was recorded in the CHCC or medical records; and
5. proportion of women with GDM for whom breastfeeding at discharge and/or provision of artificial formula is reported in the MPDC data or medical records. Analysis of secondary outcomes is being conducted on the random sample validated by medical record review (Table 3).

**Table 3:** Normal glucose parameters

Test	Glucose load	Normal reference range		Impaired glucose tolerance		Abnormal glucose tolerance	
		Pregnant	Not pregnant	Pregnant	Not pregnant	Pregnant	Not pregnant
OGTT Fasting/FPG	75 g	3.6–5.4	3.6–5.4	5.5–6.9	5.5–6.9	> = 7.0	> = 7.0
OGTT 1 h	75 g					>10.0	>10.0
OGTT 2 h	75 g	<5.5	<7.8	5.5–7.9	7.8–11.0 (Abnormal if FPG also >6.1)	>8.0	>11.0
OGCT 1 h	50 g	3.6–7.7	na		na	>7.8	
OGCT 1 h	75 g	3.6–7.9	na		na	>7.8	
HBA1C	NA	<7%					>7%
RPG	NA		<6.9		6.9–11.0		>11.00

Source: (American Association for Clinical Chemistry 2012; Colagiuri et al. 2009; Nankervis et al. 2013)

Assessment of rates of progression from GDM to T2DM is being assessed by reporting the proportion of women who have a positive diagnosis of T2DM recorded in laboratory-based tests after GDM.

### Confounding factors

A range of variables are being collected to examine a possible confounding effect on the primary outcomes, which may identify barriers or enablers to postpartum screening for T2DM. These include:

- Demographic factors: Aboriginal and Torres Strait Islander status; Maternal country of birth; Requiring a translator; Degree of remoteness of home address based on the Accessibility/Remoteness Index of Australia (ARIA) code.
- Clinical factors: Date of confinement; Pregnancy outcomes; Parity; Previous diagnosis of GDM; Gestational age of first antenatal visit and GDM diagnosis; Treatment for diabetes in pregnancy; Number of antenatal care visits; Body Mass Index; Smoking in pregnancy; Medical or pregnancy complications; Hospitalisations in pregnancy; Induction; Mode of birth; Infant birth-weight; Infant gestational age; Infant death.
- Service delivery factors: Antenatal and postnatal care provider (Hospital, Private General Practitioner, Government Health Clinic, Community Controlled Health Service).

### Indigenous status

Indigenous (Aboriginal) status is a measure of whether a person identifies as being of Aboriginal or Torres Strait Islander origin. Classification as Indigenous includes: Aboriginal but not Torres Strait Islander origin; Torres Strait Islander but not Aboriginal origin; or both Aboriginal and Torres Strait Islander origin. People classified as non-Indigenous (non-Aboriginal) are those not of Aboriginal or Torres Strait Islander origin. Recent studies suggest there is likely to be under-enumeration of Aboriginal status in population-based datasets, by up to 40% (Population and Public Health Division 2012), despite increases in the number of people identifying as Aboriginal over time (Australian Bureau of Statistics 2012).

See Additional file 1 for full list of data collected, data source, and dictionary used for collection.

### Data linkage

The security and protection of identifiable data have been an absolute priority in this study. Collection and linkage of identifiable data are being retained within secure infrastructure, to ensure the privacy of individuals. Data for women who gave birth at CH and were diagnosed with GDM have been identified in the CHCC and exported into a password protected file. This data includes the maternal first name and surname, maternal date of birth, infant date of birth, Medicare Number and GDM ICD code. This file has been sent to the Queensland Health Statistics Unit to securely provide relevant MPDC pregnancy and birth details, and to the private laboratories to provide T2DM glucose screening and diagnostic data. Separate worksheets have been developed for (1) Maternal details, with one record for each mother, including GP details for follow-up, (2) Pregnancy and birth details, with one record for each pregnancy, (3) Babies details, with one record for each baby, and (4) Postpartum screening details, with one record per screening test.

Data from CHCC and the MPDC are being combined onto worksheets and downloaded into a Microsoft Access© database for medical record review. Following medical record review, postpartum glucose test data are being assessed individually in relationship to each recorded pregnancy. If data suggests there is sub-optimal postpartum T2DM screening and further T2DM screening is recommended, letters are being sent to the woman’s primary care provider, suggesting they offer the women a screening test if their records also confirm T2DM screening has not been undertaken.

### Data analysis

De-identified data is being exported from Microsoft Access© into Stata 11© for analysis. Preliminary descriptive analysis will include assessment of the accuracy of GDM case ascertainment during data linkage and medical record review, with comparisons of rates reported in local Cairns Diabetes Centre data and the National Diabetes Services Scheme. General characteristics of mothers, pregnancies, infant’s, and screening tests are being described. All analyses are being stratified by Aboriginal status. Multiple logistic regression analysis is being used to investigate possible confounding and effect modification of variables on the primary outcomes, including confinement year to assess trends. Survival analyses and Kaplan Meir curves to assess time from confinement coded as GDM until OGTT or ‘any’ laboratory-based screening test, and time for progression from GDM to T2DM. Univariate hazards ratios will calculated using Cox proportional hazards models to assess difference between Aboriginal and non-Aboriginal women. Tests will be two tailed and p < 0.05 will be considered statistically significant.

The time frames are the date of confinement (with GDM pregnancy) to date of positive T2DM laboratory test result to estimate the mean number of months. Where the diagnosis date is not available, the date of glucose screening test which reports previous diagnosis of T2DM will be used.

### Ethics

Ethics is a primary concern for any research involving identifiable health information, particularly for Aboriginal and Torres Strait Islander people. This research was conducted in accordance with the *Guidelines for Ethical Conduct in Aboriginal and Torres Strait Islander Research* and the *Public Health Act 2005*. The research is in partnership with the Aboriginal Community Controlled Health Organisation (Apunipima Cape York Health Council) and Aboriginal researchers are included on the research team. The research was requested by clinical service providers, thereby maximising the likelihood the findings will be relevant and used for improving postpartum care services for women diagnosed with GDM in far north Queensland.

Ethical approval was granted for this project by the Cairns Hospital and Hinterland Research Ethics Committee, the Monash University Human Research and Ethics Committee (no. 201101190), and approval for accessing data under the Public Health Act granted by the Queensland Health Research Ethics and Governance Unit. Further ‘site specific applications’ were approved by the responsible authority for each department and organisation involved in collection of data, and the Queensland Health Research Ethics and Governance Unit and Cairns Hospital Research Governance Unit.

## Discussion

This retrospective cohort study is using linked administrative, registry and laboratory data, validated by medical record review, to evaluate the effectiveness of postpartum care for women diagnosed with GDM in far north Queensland from 2004 to 2010. This paper describes how existing data can be linked to evaluate existing postpartum care and examine potential barriers and enablers to postpartum care for Aboriginal and non-Aboriginal women with GDM, across different geographic localities. Furthermore, examination of case ascertainment accuracy will provide practical information on which data sources may be used to assist postpartum follow-up.

A limitation posed by this retrospective analysis is that low screening rates will underestimate the number of women who progress from GDM to T2DM. While a prospective cohort study would be a stronger study design for the prosed research questions, the case for this must be established by conducting the current study. The study also uses administrative data for case identification, which further underestimates the total number of women with GDM. However we are using several strategies to assess and improve data quality, such as data linkage and reviewing medical records. Another limitation is the inability to include ‘point of care’ tests, such as HbA1C, RPGs, and FPGs, therefore the ‘any test’ estimates in this study are likely to be lower than the true glucose screening rates. Nevertheless, OGTTs are recommended for postpartum screening in the protocols we are evaluating, as both FPG and HbA1C have lower sensitivity and will not detect isolated impaired glucose tolerance (Keely 2012; Picon et al. 2012), and we believe our reporting of rates of postpartum OGTTs in this region will be accurate.

The research is being conducted within a strong ethical framework which includes Aboriginal people on the research team, and is important to minimize the risk of harm and ensure the current research is beneficial for participants and the broader community. Service providers have requested the research and are active members of the research team, so it is highly likely the findings will be used for improving health services.

Evaluation of postpartum T2DM screening has been identified as a ‘high priority’ research need for GDM (Bennett et al. 2012); particularly for Aboriginal women who are at high risk for GDM and subsequent T2DM, and are the subject of recent recommendations for increased GDM screening in early pregnancy. These changes to screening during pregnancy will be of little benefit for Aboriginal women unless effective prevention, treatment and postpartum care are provided. This research is urgently needed to improve postpartum care for Aboriginal women with GDM, to mitigate the risks to women, subsequent children, and future generations, and to reduce health disparities experienced by Aboriginal peoples.

## Endnote

^a^The term ‘Aboriginal’ is used when referring specifically to Aboriginal and Torres Strait Islander people in Australia, and the term ‘Indigenous’ is used when referring more generally to Indigenous people’s worldwide. This is for ease of reading in this paper only, and we respectfully acknowledge the diversity and autonomy of Indigenous peoples, including Torres Strait Islander people.

## Authors’ information

CC is undertaking this research as part of a PhD program, supported by a National Health and Medical Research Council PhD scholarship (607247) and a Population Health Capacity Building Grant (457379). The need for this research project to evaluate existing services was identified by staff at the Cairns Diabetes Centre, and the gap in evidence for postpartum care for Indigenous women with GDM was identified in a comprehensive systematic review (Chamberlain et al. 2013).

## Electronic supplementary material

Additional file 1: Data Dictionary. (DOCX 21 KB)

## Abbreviations

ARIA: Accessibility/Remoteness index of Australia
CH: Cairns hospital
CHCC: Cairns base hospital clinical coding
DIP: Diabetes in pregnancy
FPG: Fasting plasma glucose
GDM: Gestational diabetes mellitus
HbA1C: Glycosated haemoglobin
IADPSG: International association for diabetes in pregnancy study group
ICD: International classification of disease
IRRA: Indigenous research reform agenda
MPDC: Midwives perinatal data collection
NDSS: National diabetes services scheme
OGTT: Oral glucose tolerance test
PAG: Project advisory group
PHIS: Primary healthcare information system
RPG: Random plasma glucose
SES: Socioeconomic status
T2DM: Type 2 diabetes mellitus
US: United States.

## References

[CR1] American Association for Clinical Chemistry (2012). Lab tests online.

[CR2] American Diabetes Association (2009). Diagnosis and classification of diabetes mellitus (position statement). Diabetes Care.

[CR3] Australian Bureau of Statistics (2008). Diabetes in the aboriginal and Torres Strait Islander population.

[CR4] Australian Bureau of Statistics (2010). The health and welfare of Australia’s aboriginal and Torres Strait Islander peoples, 2010.

[CR5] Australian Bureau of Statistics (2012). Information paper: perspectives on aboriginal and Torres Strait Islander identification in selected data collection contexts.

[CR6] Australian Health Ministers’ Advisory Council (2008). Population based screening framework.

[CR7] Australian Institute of Health and Welfare (2010). Australia’s health 2010.

[CR8] Australian Institute of Health and Welfare (2010). Diabetes in pregnancy: its impact on Australian women and their babies. Diabetes series no. 14.

[CR9] Australian Institute of Health and Welfare (2012). National Data Dictionary Version 2012.

[CR10] Bell R, Lie M, Hayes L, Lewis-Barned N, May C, White M (2011). Preventing Type 2 diabetes after gestational diabetes: Women’s experiences and implications for diabetes prevention interventions. Diabet Med.

[CR11] Bellamy L, Casas J-P, Hingorani AD, Williams D (2009). Type 2 diabetes mellitus after gestational diabetes: a systematic review and meta-analysis. Lancet.

[CR12] Benjamin E, Winters D, Mayfield J, Gohdes D (1993). Diabetes in pregnancy in Zuni Indian women. Prevalence and subsequent development of clinical diabetes after gestational diabetes. Diabetes Care.

[CR13] Bennett WL, Robinson KA, Saldanha IJ, Wilson LM, Nicholson WK (2012). High priority research needs for gestational diabetes mellitus. J Womens Health.

[CR14] Bentley-Lewis R, Levkoff S, Stuebe A, Seely EW (2008). Gestational diabetes mellitus: postpartum opportunities for the diagnosis and prevention of type 2 diabetes mellitus. Nat Clin Pract Endocrinol Metab.

[CR15] Bhattarai MD (2009). Three patterns of rising type 2 diabetes prevalence in the world: Need to widen the concept of prevention in individuals into control in the community. JNMA J Nepal Med Assoc.

[CR16] Bilhartz T, Bilhartz P, Bilhartz T, Bilhartz R (2011). Making Use of a Natural Stress Test: Pregnancy and Cardiovascular Risk. J Womens Health.

[CR17] Bower C, Stanley F, Connell AF, Gent CR, Massey MS (1992). Birth defects in the infants of aboriginal and non-aboriginal mothers with diabetes in Western Australia. Med J Aust.

[CR18] Brown W, Trost S (2003). Life transitions and changing physical activity patterns in young women. Am J Prev Med.

[CR19] Canadian Diabetes Association (2011). Diabetes: Canada at the tipping point—charting a new path.

[CR20] Carson MP, Frank MI, Keely E (2013). Postpartum testing rates among women with a history of gestational diabetes: Systematic review. Prim Care Diabetes.

[CR21] Chamberlain C, Yore D, Li H, Williams E, Oldenburg B, Oats J, McNamara B, Eades S (2011). Diabetes in pregnancy among indigenous women in Australia, Canada, New Zealand, and the United States: a method for systematic review of studies with different designs. BMC Pregnancy Childbirth.

[CR22] Chamberlain C, McNamara B, Williams ED, Yore D, Oldenburg B, Oats J, Eades S (2013). Diabetes in pregnancy among indigenous women in Australia, Canada, New Zealand and the United States: A systematic review of the evidence for screening in early pregnancy. Diabetes Metab Res Rev.

[CR23] Chodick G, Elchalal U, Sella T, Heymann A, Porath A, Kokia E, Shalev V (2010). The risk of overt diabetes mellitus among women with gestational diabetes: a population-based study. Diabet Med.

[CR24] Clark HD, Keely E (2012). Getting mothers with gestational diabetes to return for postpartum testing: What works and what does not. Diabetes Manag.

[CR25] Colagiuri S, Davies D, Girgis S, Colagiuri R (2009). National evidence based guideline for case detection and diagnosis of Type 2 diabetes.

[CR26] Cosson E (2010). Diagnostic criteria for gestational diabetes mellitus. Diabetes Metab.

[CR27] Coustan DR (2010). Finding and treating gestational diabetes mellitus–does it help?. Nat Rev Endocrinol.

[CR28] Coustan DR, Lowe LP, Metzger BE, Dyer AR (2010). The Hyperglycaemia and Adverse Pregnancy Outcome (HAPO) study: paving the way for new diagnostic criteria for gestational diabetes mellitus. Am J Obstet Gynecol.

[CR29] Crowther C, Hiller J, Moss J, McPhee A, Jeffries W, Robinson J (2005). Australian carbohydrate intolerance study in pregnant women (ACHOIS) trial group: Effect of treatment of gestational diabetes mellitus on pregnancy outcomes. N Engl J Med.

[CR30] Daniells S, Grenyer B, Davis W, Coleman K, Burgess J, Moses R (2003). Gestational diabetes mellitus: is a diagnosis assoicated with an increase in maternal anxiety and stress in the short and intermediate term?. Diabetes Care.

[CR31] Dannenbaum D, Verronneau M, Torrie J, Smeja H, Robinson E, Dumont C, Kovitch I, Webster T (1999). Comprehensive computerized diabetes registry. Serving the Cree of Eeyou Istchee (eastern James Bay). Can Fam Physician.

[CR32] Davis B, Bond D, Howat P, Sinha AK, Falhammar H, Davis B, Bond D, Howat P, Sinha AK, Falhammar H (2009). Maternal and neonatal outcomes following diabetes in pregnancy in Far North Queensland, Australia. Aust N Z J Obstet Gynaecol.

[CR33] Davis B, McLean A, Sinha AK, Falhammar H (2013). A threefold increase in gestational diabetes over two years: Review of screening practices and pregnancy outcomes in Indigenous women of Cape York, Australia. Aust N Z J Obstet Gynaecol.

[CR34] Demirtas B (2012). Strategies to support breastfeeding: a review. Int Nurs Rev.

[CR35] Depczynski B, Wong VW, Russell HD, Opi N (2011). The impact of potential new diagnostic criteria on the prevalence of gestational diabetes mellitus in Australia. Med J Aust.

[CR36] Dyck RF (2005). Tracking ancient pathways to a modern epidemic: diabetic end-stage renal disease in Saskatchewan aboriginal people. Kidney Int Suppl.

[CR37] Dyck RF, Sheppard MS, Cassidy H, Chad K, Tan L, Van Vliet SH (1998). Preventing NIDDM among aboriginal people: is exercise the answer? Description of a pilot project using exercise to prevent gestational diabetes. Int J Circumpolar Health.

[CR38] Dyck R, Osgood N, Lin TH, Gao A, Stang MR (2010). Epidemiology of diabetes mellitus among First Nations and non-First Nations adults. Can Med Assoc J.

[CR39] Eades SJ, Taylor B, Bailey S, Williamson AB, Craig JC, Redman S, for the SEARCH Investigators (2010). The health of urban Aboriginal people: insufficient data to close the gap. Med J Aust.

[CR40] Falhammar H, Davis B, Sinha A (2010). Maternal and neonatal outcomes in the Torres Strait with a sixfold increase in type 2 diabetes in pregnancy over six years. Aust N Z J Obstet Gynaecol.

[CR41] Farrell T, Neale L, Cundy T (2002). Congenital anomalies in the offspring of women with type 1, type 2 and gestational diabetes. Diabet Med.

[CR42] Flack J, Ross G, Ho S, McElduff A (2010). Recommended changes to diagnostic criteria for gestational diabetes: Impact on workload. Aust N Z J Obstet Gynaecol.

[CR43] Gabbe SG, Landon MB, Warren-Boulton E, Fradkin J (2011). Promoting health after gestational diabetes: A national diabetes education program call to action. Obstet Gynecol.

[CR44] Gray-Donald K, Robinson E, Collier A, David K, Renaud L, Rodrigues S (2000). Intervening to reduce weight gain in pregnancy and gestational diabetes mellitus in Cree communities: an evaluation. Can Med Assoc J.

[CR45] HAPO Study Cooperative Research Group (2008). Hyperglycaemia and Adverse Pregnancy Outcomes. N Engl J Med.

[CR46] Heikes KE, Eddy DM, Arondekar B, Schlessinger L (2008). Diabetes Risk Calculator: A simple tool for detecting undiagnosed diabetes and pre-diabetes. Diabetes Care.

[CR47] Hoffman L, Nolan C, Wilson DA, Oats J, Simmons D (1998). Gestational diabetes mellitus - management guidelines. Med J Aust.

[CR48] Hunt KJ, Schuller KL (2007). The increasing prevalence of diabetes in pregnancy. Obstet Gynecol Clin North Am.

[CR49] International Association of Diabetes and Pregnancy Study Groups (2010). International Association of Diabetes and Pregnancy Study Groups Recommendations on the Diagnosis and Classification of Hyperglycaemia in Pregnancy. Diabetes Care.

[CR50] Jones EJ, Appel SJ, Eaves YD, Moneyham L, Oster RA, Ovalle F (2012). Cardiometabolic risk, knowledge, risk perception, and self-efficacy among American Indian women with previous gestational diabetes. J Obstet Gynecol Neonatal Nurs.

[CR51] Kalra S, Malik S, John M (2011). Gestational diabetes mellitus: A window of opportunity. Indian J Endocrinol Metab.

[CR52] Karanja N, Lutz T, Ritenbaugh C, Maupome G, Jones J, Becker T, Aickin M (2010). The TOTS Community Intervention to Prevent Overweight in American Indian Toddlers Beginning at Birth: A Feasibility and Efficacy Study. J Community Health.

[CR53] Keely E (2012). An opportunity not to be missed–how do we improve postpartum screening rates for women with gestational diabetes?. Diabetes Metab Res Rev.

[CR54] Keely E, Clark H, Karovitch A, Graham I (2010). Screening for type 2 diabetes following gestational diabetes: family physician and patient perspectives. Can Fam Physician.

[CR55] Kim C (2002). Gestational diabetes and the incidence of type 2 diabetes: a systematic review. Diabetes Care.

[CR56] Kim C (2007). Risk perception for diabetes among women with histories of gestational diabetes mellitus. Diabetes Care.

[CR57] Klomp H, Dyck R, Sheppard S (2003). Description and evaluation of a prenatal exercise program for urban aboriginal women. Can J Diabetes.

[CR58] Knowler WC (2002). Reduction in the incidence of type 2 diabetes with lifestyle intervention or metformin. N Engl J Med.

[CR59] Korpi-Hyovalti E, Laaksonen DE, Schwab U, Heinonen S, Niskanen L (2012). How can we increase postpartum glucose screening in women at high risk for gestational diabetes mellitus?. Int J Endocrinol.

[CR60] Landon M, Spong C, Thom E (2009). A multicenter, randomized trial of treatment for mild gestational diabetes. N Engl J Med.

[CR61] Langer N, Langer O (1994). Emotional adjustment to diagnosis and intensified treatment of gestational diabetes. Obstet Gynecol.

[CR62] Laws P, Sullivan EA (2004). Australia’s mothers and babies 2001.

[CR63] Lee AJ, Hiscock RJ, Wein P, Walker SP, Permezel M (2007). Gestational Diabetes Mellitus: Clinical Predictors and Long-Term Risk of Developing Type 2 Diabetes: A retrospective cohort study using survival analysis. Diabetes Care.

[CR64] Lega IC, McLaughlin H, Coroneos M, Handley-Derry F, Donovan N, Lipscombe LL (2011). A physician reminder to improve postpartum diabetes screening in women with gestational diabetes mellitus. Diabetes Res Clin Pract.

[CR65] Leiberman N, Kalter-Leibovici O, Hod M (2011). Global adoption of IADSPG recommendations: A national approach. Int J Gynaecol Obstet.

[CR66] Lindsay RS (2011). Gestational diabetes: costs and consequences. Diabetologica.

[CR67] Liu B, Jorm L, Banks E (2010). Parity, breastfeeding, and the subsequent risk of maternal type 2 diabetes. Diabetes Care.

[CR68] McBride CM, Emmons KM, Lipkus IM (2003). Understanding the potential of teachable moments: the case of smoking cessation. Health Educ Res.

[CR69] McGrath NM, Evans C, Holgate A (2007). Post-partum follow-up of women with gestational diabetes mellitus from Northland, New Zealand. Diabet Med.

[CR70] McNamara BJ, Sanson-Fisher R, D’Este C, Eades S (2011). Type 2 diabetes in Indigenous populations: Quality of intervention research over 20 years. Prev Med.

[CR71] Metcalfe AR (2012). Maternal morbidity data in Australia: an assessment of the feasibility of standardised collection.

[CR72] Mohamed N, Dooley J (1998). Gestational diabetes and subsequent development of NIDDM in aboriginal women of northwestern Ontario. Int J Circumpolar Health.

[CR73] Moran LJ, Lombard CB, Lim S, Noakes M, Teede HJ (2010). Polycystic ovary syndrome and weight management. (Report). Womens Health.

[CR74] Morikawa M, Yamada T, Yamada T, Akaishi R, Nishida R, Cho K (2010). Changes in the number of patients after the adoption of IADSPG criteria for hyperglycaemia during pregnancy in Japanese women. Diabetes Res Clin Pract.

[CR75] Morrison MK, Collins CE, Lowe JM (2009). Postnatal testing for diabetes in Australian women following gestational diabetes mellitus. Aust N Z J Obstet Gynaecol.

[CR76] Moses R, Morris G, Petocz P, San Gil F, Garg D (2011). The impact of potential new diagnostic criteria on the prevalence of gestational diabetes mellitus in Australia. Med J Aust.

[CR77] Murphy S, Wilson C (2008). Breastfeeding promotion: a rational and achievable target for a type 2 diabetes prevention intervention in Native American communities. J Hum Lact.

[CR78] Nankervis A, McIntyre HD, Moses R, Ross GP, Callaway L, Porter C, Jeffries W, Boorman C, De Vries B (2013). Consensus guidelines for the testing and diagnosis of gestational diabetes in Australia.

[CR79] Naqshbandi M, Harris S, Esler J, Antwi-Nsiah F (2008). Global complication rates of type 2 diabetes in Indigenous peoples: A comprehensive review. Diabetes Res Clin Pract.

[CR80] Neufeld H (2011). Food Perceptions and Concerns of Aboriginal Women Coping with Gestational Diabetes in Winnipeg, Manitoba. J Nutr Educ Behav.

[CR81] O’Dea K, Rowley K, Brown A (2007). Diabetes in Indigenous Australians: Possible ways forward. Med J Aust.

[CR82] O’Reilly M, Avalos G, Dennedy M, O’Sullivan E, Dunne F (2011). Atlantic DIP: high prevalence of abnormal glucose tolerance post partum is reduced by breast-feeding in women with prior gestational diabetes mellitus. Eur J Endocrinol.

[CR83] O’Sullivan E, Avalos G, O’Reilly M, Dennedy M, Gaffney G, Dunne F, on behalf of the Atlantic DIP collaborators (2011). Atlantic Diabetes in Pregnancy (DIP): the prevalence and outcomes of gestational diabetes mellitus using new diagnostic criteria. Diabetologica.

[CR84] Orleans CT, Barker DC, Kaufman NJ, Marx JF (2000). Helping pregnant smokers quit: meeting the challenge in the next decade. Tob Control.

[CR85] Osgood ND, Dyck RF, Grassmann WK (2011). The inter- and intragenerational impact of gestational diabetes on the epidemic of type 2 diabetes. Am J Public Health.

[CR86] Owen CG, Martin RM, Whincup PH, Smith GD, Cook DG (2006). Does breastfeeding influence risk of type 2 diabetes in later life? A quantitative analysis of published evidence. Am J Clin Nutr.

[CR87] Pan XR (1997). Effects of diet and exercise in preventing NIDDM in people with impaired glucose tolerance. The Da Qing IGT and Diabetes Study. Diabetes Care.

[CR88] Pettitt DJ, Forman MR, Hanson RL, Knowler WC, Bennett PH (1997). Breastfeeding and incidence of non-insulin-dependent diabetes mellitus in Pima Indians. Lancet.

[CR89] Picon MJ, Murri M, Munoz A, Fernandez-Garcia JC, Gomez-Huelgas R, Tinahones FJ (2012). Hemoglobin A1c versus oral glucose tolerance test in postpartum diabetes screening. Diabetes Care.

[CR90] Pierce M, Modder J, Mortagy I, Springett A, Hughes H, Baldeweg S (2011). Missed opportunities for diabetes prevention: Post-pregnancy follow-up of women with gestational diabetes mellitus in England. Br J Gen Pract.

[CR91] Population and Public Health Division (2012). Improved reporting of Aboriginal and Torres Strait Islander peoples on population datasets in New South Wales using record linkage–a feasibility study.

[CR92] Queensland Health (2008). Cairns base hospital and associated services Clinical Services Plan.

[CR93] Queensland Health and Royal Flying Doctors Service (Queensland section) (2005). Primary clinical care manual.

[CR94] Queensland Health and Royal Flying Doctors Service (Queensland section) (2007). Primary clinical care manual.

[CR95] Queensland Health and Royal Flying Doctors Service (Queensland Section) (2009). Primary clinical care manual.

[CR96] Razee H, van der Ploeg HP, Blignault I, Smith BJ, Baumann AE, McLean M, Cheung NW (2010). Beliefs, barriers, social support, and environmental influences related to diabetes risk behaviours among women with a history of gestational diabetes. Health Promot J Austr.

[CR97] Round JA, Jacklin PB, Fraser RB, Hughes RG, Mugglestone MA, Holt RIG (2010). Screening for gestational diabetes mellitus: cost-utility of different screening strategies based on a woman’s individual risk of disease. Diabetologica.

[CR98] Rumbold A, Crowther C (2002). Women’s experiences of being screened for gestational diabetes mellitus. Aust N Z J Obstet Gynaecol.

[CR99] Rumbold AR, Bailie RS, Si D, Dowden MC, Kennedy CM, Cox RJ, O’Donoghue L, Liddle HE, Kwedza RK, Thompson SC, Burke HP, Brown A, Weeramanthri T, Connors CM (2011). Delivery of maternal health care in Indigenous primary care services: baseline data for an ongoing quality improvement initiative. BMC Pregnancy Childbirth.

[CR100] Russell MA (2006). Rates of postpartum glucose testing after gestational diabetes mellitus. Obstet Gynecol.

[CR101] Sable MJ, Wilkinson DS (2000). Impact of Perceived Stress, Major Life Events And Pregnancy Attitudes on Low Birth Weight. Fam Plann Perspect.

[CR102] Schwarz EB, Brown JS, Creasman JM, Stuebe A, McClure CK, Van Den Eeden SK, Thom D (2010). Lactation and maternal risk of type 2 diabetes: a population-based study. Am J Med.

[CR103] Shah BR, Lipscombe LL, Feig DS, Lowe JM (2011). Missed opportunities for type 2 diabetes testing following gestational diabetes: A population-based cohort study. BJOG.

[CR104] Sicree R, Shaw J, Zimmet P (2009). The Global Burden Diabetes and Impaired Glucose Tolerance. Diabetes.

[CR105] Simmons D, Khan MA, Teale G, Simmons D, Khan MA, Teale G (2005). Obstetric outcomes among rural Aboriginal Victorians. Aust N Z J Obstet Gynaecol.

[CR106] Smirnakis KV (2005). Postpartum diabetes screening in women with a history of gestational diabetes. Obstet Gynecol.

[CR107] Stasenko M, Liddell J, Cheng YW, Sparks TN, Killion M, Caughey AB (2011). Patient counseling increases postpartum follow-up in women with gestational diabetes mellitus. Am J Obstet Gynecol.

[CR108] Steinhart JR, Sugarman JR, Connell FA (1997). Gestational diabetes is a herald of NIDDM in Navajo women. High rate of abnormal glucose tolerance after GDM. Diabetes Care.

[CR109] Stephenson J (1993). Diabetes in the Aboriginal Community. Aborig Isl Health Work J.

[CR110] Sterne VL, Logan T, Palmer MA (2011). Factors affecting attendance at postpartum diabetes screening in women with gestational diabetes mellitus. Pract Diab Int.

[CR111] Taylor LK, Travis S, Pym M, Olive E, Henderson-Smart DJ (2005). How useful are hospital morbidity data for monitoring conditions occurring in the perinatal period?. Aust N Z J Obstet Gynaecol.

[CR112] Teh WT, Teede HJ, Paul E, Harrison CL, Wallace EM, Allan C (2011). Risk factors for gestational diabetes mellitus: Implications for the application of screening guidelines. Aust N Z J Obstet Gynaecol.

[CR113] Tovar A, Chasan-Taber L, Eggleston E, Oken E (2011). Postpartum screening for diabetes among women with a history of gestational diabetes mellitus. Prev Chronic Dis.

[CR114] Tuomilehto J (2001). Prevention of type 2 diabetes mellitus by changes in lifestyle among subjects with impaired glucose tolerance. N Engl J Med.

[CR115] van der Pligt P, Willcox J, Hesketh KD, Ball K, Wilkinson S, Crawford D, Campbell K (2013). Systematic review of lifestyle interventions to limit postpartum weight retention: implications for future opportunities to prevent maternal overweight and obesity following childbirth. Obes Rev.

[CR116] Wikipedia (2013). Far North Queensland.

[CR117] Wilson J, Jungner G (1968). Principles and practice of screening for disease. Public health paper number 34.

[CR118] Young TK, Martens PJ, Taback SP, Sellers EA, Dean HJ, Cheang M, Flett B, Young TK, Martens PJ, Taback SP, Sellers EAC, Dean HJ, Cheang M, Flett B (2002). Type 2 diabetes mellitus in children: prenatal and early infancy risk factors among native canadians. Arch Pediatr Adolesc Med.

[CR119] Yue DK, Molyneaux LM, Ross GP, Constantino MI, Child AG, Turtle JR (1996). Why does ethnicity affect prevalence of gestational diabetes? The underwater volcano theory. Diabet Med.

