# Supplementary material for: Postpartum care for Aboriginal and non-Aboriginal women with Gestational Diabetes Mellitus across urban, rural and remote locations: a protocol for a cohort linkage study
Source: Springerplus. 2013 Oct 30;2:576. doi: 10.1186/2193-1801-2-576 (PMC4320232; doi:10.1186/2193-1801-2-576)
Supplement: Supplementary file 1 — Additional file 1: Data Dictionary. (DOCX 21 KB) [file 40064_2013_1430_MOESM1_ESM.docx]

**Additional file 1: Data Dictionary**

| **Data item description** | **Response options** |
| --- | --- |
| Mother's surname | Surname |
| Mother's first name | Firstname |
| Mother's Date of birth | YYYYMMDD |
| Aboriginal or Torres Strait Islander origin | 1=Ab, 2=TSI, 3=both, 4=neither, 9=not stated/unknown |
| Mother's country of birth | 4 digit ASCCSS country code |
| English a second language (needs translator) | Requires translator as first language other than English: 1= no, 2=yes, 9= unknown |
| Surname of mother's GP | Surname |
| Firstname of mother's GP | First name |
| GP Street address | Street number and name |
| Suburb/town of GP | suburb/town name |
| State GP located | State |
| Postcode of GP |  |
| Phone number of GP | 10 digit phone number (including area code) |
| Date of confinement Corresponds to date of birth (or first baby in multiple births) | YYYYMMDD |
| Baby outcome in pregnancy | 1=single liveborn, 2=single stillborn, 3=twins-bothliveborn, 4=twins - one live one still born, 5=other multiple-both liveborn, 9=unknown |
| Diabetes in pregnancy: Any diabetes | 1= no, 2=yes, 9=unknown |
| Gestational diabetes: Diabetes first recognised during pregnancy (not diagnosed prior to pregnancy) | 1= no, 2=yes, 9=unknown |
| GDM diagnosed in a previous pregnancy | 1= no, 2=yes, 9=unknown |
| If previous GDM, diagnosed date: (medical record review-01/01 if unknown MMDD) | YYYYMMDD |
| Gestational age diagnosed with GDM this pregnancy | 2 digit no.- completed weeks pregnancy |
| Date diagnosed with GDM | YYYYMMDD |
| Maternal diabetes Treatment in pregnancy | 1=none, 2=insulin, 3=oral med, 4=other, 9= unknown or not stated |
| Maternal diabetes Treatment in pregnancy | 1=none, 2=insulin, 3=oral med, 4=other, 9= unknown or not stated |
| Maternal diabetes Treatment in pregnancy | 1=none, 2=insulin, 3=oral med, 4=other, 9= unknown or not stated |
| Postcode | 4 digit postcode |
| 4 digit ASCSS code: degree of remoteness | Aria code |
| Marital status | 1=never married, 2=married/defacto, 3=widowed, 4=divorced, 5=separated, 9=unknown |
| Location of birth | Location of birth: Cairns Base Hospital(1), Thursday Island Hospital(2), Other (3), Unknown (9) |
| Maternal length of stay | 2 or 3 digit number |
| Any antenatal care | 1=no, 2=yes, 9=unknown |
| Number of antenatal visits for this pregnancy | 1=less than 2, 3=2-4, 4=5-7, 5=8 or more, 9=not stated/unknown |
| Location of antenatal care | 1=none, 2=CBH,3= Private GP clinic, 4=government health clinic, 5=community controlled health service, 6=other (including mixed), 9=unknown |
| Gestational age of first antenatal visit | 2 digit number completed weeks gestation |
| Previous number of births (>20 weeks gestation) | 2 digit number |
| No. births during 2004-2010 | 2 digit number |
| BMI at first antenatal visit | calculate wt/ht |
| Maternal Weight at first antenatal visit | 3 num |
| Maternal height at first antenatal visit | 3 num |
| Cigarette smoking prior to 20 weeks pregnancy | 1=no, 2=yes, 9=unknown |
| Continued smoking after 20 weeks pregnancy | 1=no, 2=yes, 9=unknown |
| Medical complications affecting pregnancy | 1=no, 2=yes, 9=unknown. |
| Pregnancy complications in pregnancy (pre-eclampsia, gestational hypertension etc) | 1=no, 2=yes, 9=unknown. |
| Hospitalization during pregnancy | 1=no, 2=yes, 9=unknown |
| If hospitalised in pregnancy , reason | description |
| Referral to dietician | 1=no, 2=yes, 9=unknown |
| Number of dietician visits | 2 digit number |
| Referral to diabetes educator | 1=no, 2=yes, 9=unknown |
| If yes, number Diabetes Educator visits | 2 digit number |
| Other interventions to reduce risk of T2DM for mother | 1=none, 2=group education, 3=other, 9=unknown |
| If OtherPrev=3, describe | Brief description |
| Induction | 1=no, 2=yes, 9=unknown |
| If induced, state indication | Describe |
| Mode of birth | 10=v, 02=F, 3=VE, 04=Luscs, 05=cl cs, 98=other, 99=unknown |
| Breastfeeding Interventions described to reduce risk of T2DM for baby | 1=no, 2=antenatal expressing, 3=other, 9=unknown. |
| If BFInt=3(Other), describe | Describe intervention briefly |
| Breastfeeding at discharge | 1=no, 2=ever but ceased, 3=any/partial on d/c, 4= all, 9=unknown) |
| Location of postnatal care | 1= none, 2=CBH, 3= Private GP clinic, 4= government health clinic, 5=community controlled health service, 6=other, 9=unknown |
| Postnatal interventions described to reduce risk of T2DM for mother | 1=none, 2=diet and exercise advice, 3= group education, 4=other, 9=unknown |
| If PNInt=4(Other), describe | Brief description |
| Baby Born alive | 1=alive, 2=stillborn, 9=unknown |
| Date of baby's birth | YYYYMMDD |
| Baby's Birthweight (g) | 4 digits(g) |
| Gestational age of baby at birth (completed weeks) | 2 digits |
| Baby admitted to | 1=no, 2=yes, 9=unknown |
| If admitted number days in SCN | 3 digit number |
| Indication for SCN admission: 5 digit ICD10 code | 5 digit number |
| Baby hypoglycaemia | 1=no, 2=yes, 9=unknown |
| Baby born with congenital anomaly | 1=no, 2=yes, 3=suspected, 9=unknown |
| Baby given artificial formula prior to discharge | 1=no, 2=yes, 9=unknown |
| If AF=2: Indication for Artificial formula in hospital | Brief description |
| T2DM screening/ diagnostic followup test provided | 1=n, 2=y , 9=unknown |
| Date of followup | YYYYMMDD |
| Type of screening/diagnostic test | 1=none, 2=OGTT, 3=OGCT, 4=HBA1C, 5=FPG, 6=RPG, 7=Other, 9=unknown |
| If FuScrType=7, describe | Brief description of test type |
| 2h OGTT result | 3 digit number (1 decimal point) or 0 |
| FPG result - either after OGTT or just fasting (based on previous identification of OGTT as test type) | 3 digit number (1 decimal point) or 0 |
| HbA1c result | 3 digit number (1 decimal point) or 0 |
| RPG result | 4 digit number (1 decimal point) or 0 |
| Other result | 4 digit number (1 decimal point) or 0 |
| T2DM diagnosis | 1=no, 2=yes, 9=unknown |
| Date of T2DM diagnosis | YYYYMMDD |
